# Web-Based Intervention to Reduce Substance Abuse and Depressive Symptoms in Mexico: Development and Usability Test

**DOI:** 10.2196/mental.6001

**Published:** 2016-09-29

**Authors:** Marcela Tiburcio, Ma Asunción Lara, Araceli Aguilar Abrego, Morise Fernández, Nora Martínez Vélez, Alejandro Sánchez

**Affiliations:** ^1^ Ramón de la Fuente Muñiz, National Institute of Psychiatry Department of Social Sciences in Health Direction of Epidemiological and Psychosocial Research Mexico City Mexico; ^2^ Ramón de la Fuente Muñiz, National Institute of Psychiatry Department of Intervention Models Direction of Epidemiological and Psychosocial Research Mexico City Mexico; ^3^ Health Sciences Institute Universidad Veracruzana Xalapa, Veracruz Mexico

**Keywords:** substance abuse, depressive symptoms, Internet, cognitive behavioral therapy, usability

## Abstract

**Background:**

The development of Web-based interventions for substance abuse in Latin America is a new field of interest with great potential for expansion to other Spanish-speaking countries.

**Objective:**

This paper describes a project aimed to develop and evaluate the usability of the Web-based Help Program for Drug Abuse and Depression (Programa de Ayuda para Abuso de Drogas y Depresión, PAADD, in Spanish) and also to construct a systematic frame of reference for the development of future Web-based programs.

**Methods:**

The PAADD aims to reduce substance use and depressive symptoms with cognitive behavioral techniques translated into Web applications, aided by the participation of a counselor to provide support and guidance. This Web-based intervention includes 4 steps: (1) My Starting Point, (2) Where Do I Want to Be? (3) Strategies for Change, and (4) Maintaining Change. The development of the program was an interactive multistage process. The first stage defined the core structure and contents, which were validated in stage 2 by a group of 8 experts in addiction treatment. Programming of the applications took place in stage 3, taking into account 3 types of end users: administrators, counselors, and substance users. Stage 4 consisted of functionality testing. In stage 5, a total of 9 health professionals and 20 drug users currently in treatment voluntarily interacted with the program in a usability test, providing feedback about adjustments needed to improve users’ experience.

**Results:**

The main finding of stage 2 was the consensus of the health professionals about the cognitive behavioral strategies and techniques included in PAADD being appropriate for changing substance use behaviors. In stage 5, the health professionals found the functionalities easy to learn; their suggestions were related to the page layout, inclusion of confirmation messages at the end of activities, avoiding “read more” links, and providing feedback about every activity. On the other hand, the users said the information presented within the modules was easy to follow and suggested more dynamic features with concrete instructions and feedback.

**Conclusions:**

The resulting Web-based program may have advantages over traditional face-to-face therapies owing to its low cost, wide accessibility, anonymity, and independence of time and distance factors. The detailed description of the process of designing a Web-based program is an important contribution to others interested in this field. The potential benefits must be verified in specific studies.

**Trial Registration:**

International Standard Randomized Controlled Trial Number (ISRCTN): 25429892; http://www.controlled-trials.com/ISRCTN25429892 (Archived by WebCite at http://www.webcitation.org/6ko1Fsvym)

## Introduction

### Internet-Based Cognitive Behavioral Interventions for Drug Use

The drug treatments with the greatest empirical validity are those involving cognitive behavioral interventions (CBIs) [1]. Some authors argue that the success of such interventions is related to the use of specific techniques, such as exploration of the positive and negative consequences of substance use (decisional balance); self-monitoring, or diary of use, where situations of high risk for drug use can also be identified; elaboration of strategies to anticipate and face situations of risk and craving; and training in social abilities [2-4].

A recent meta-analysis of the effectiveness of Internet-based interventions based on cognitive behavioral therapy demonstrated a large effect size (0.83, n=3960) as compared with other modalities, including psychoeducation (0.46, n=6796) [5]. On the other hand, it has been reported that the principles and techniques of CBIs focused on reduction in alcohol and tobacco use lend themselves more easily to adaption to an Internet-based format [6].

Web-based interventions allow complex treatments to be delivered with consistency and minimal demands on staff time and training resources. Moreover, computerized programs may be less threatening, provide greater anonymity [7,8], and reduce the effects of stigma, allowing individuals to seek information in relative privacy [9]. The expansion of the Internet offers new treatment opportunities for a large number of individuals at a relatively low cost; they may be particularly useful in rural or remote settings, where access to psychotherapy for substance use disorders may be limited, and they may thus help to broaden the availability of treatment [10-15]. Current Web-based programs differ in the level of therapist support provided and the use of tools that require or not a response from the user. The level of support can vary from nonassistance (self-help) to having some level of therapist contact by email or telephone; the latter has shown results superior to those of total self-help programs [5,16-18].

During the last decade, several Web-based interventions have been developed and validated in the mental health field [5]. Those for substance abuse, however, have focused mainly on alcohol [19-21] and tobacco [22,23]. There are few validated Web-based interventions to address drug abuse [24-27] and even fewer that address substance abuse and depression together even though comorbidity is highly frequent [28,29].

### Internet Use and Web-Based Treatment in Latin America

Web-based interventions developed in Latin America are very limited and focus on smoking [30], heavy drinking [31,32], and depression [33,34]. Given the 560 million people worldwide who speak Spanish, 40 million of whom live in the United States, there is a pressing need for Web-based interventions in this language [35].

Approximately 3.3 billion people around the world use the Internet, among them 345 million in Latin America, with a growth of 1808.4% in the past 15 years [36]. According to the Mexican Internet Association [37], there are approximately 53.9 million Internet users in the country, 46% of whom are aged 13-24 years. Although there are no specific studies of the use of the Internet for health care, it has been documented that health-related pages occupy the eleventh place among Web searches as a whole [38].

There are few publications that describe the process of developing a Web-based intervention, the selection of strategies required, or the limitations involved in starting it up [39]. Such information could help to provide better interpretations of data on the effectiveness of Web-based programs, aid in the design of outreach strategies to target populations, and suggest avenues for further research [40]. It could also testify to the complexity of designing such programs in a Latin American context, where they have seldom been attempted and where there is still a degree of resistance to their use.

The purposes of this paper are therefore to describe the development process of the Web-based Help Program for Drug Abuse and Depression (Programa de Ayuda para Abuso de Drogas y Depresión, PAADD, in Spanish) [41] in Mexico and to describe its final structure and functioning, which includes the participation of a counselor.

## Methods

### Stages of Development

The PAADD was developed in an interactive multistage process that involved design, testing, and redesign tasks, following international recommendations for the development of eHealth strategies and ethical standards for Web-based interventions [42].

#### Stage 1. Conceptual Design

The aims of this phase were to define the structure and contents of the program. After a search of the literature on treatment in Mexico for problems of substance use and depression, 3 sources were chosen: (1) self-help manual ¿Cómo dejar de consumir drogas? (How to Stop Using Drugs) [43]; (2) Web-based program Ayuda para Depresión (ADep, Help for Depression) [33,34]; and (3) Web-based program Beber Menos (Drink Less) [31].

The basic structure of the intervention and the specific techniques of behavior modification were taken from the self-help manual How to Stop Using Drugs [43], a brief CBI.

The Web-based program ADep [33,34] is a CBI-based self-help program addressed to women but also used by men. It was designed for the general population to reduce depressive symptoms or their severity in those already suffering from depression. It provided the basis for the cognitive restructuring component to change negative thoughts associated with substance use, as well as relaxation exercises; it does not address substance abuse.

The Web-based program Beber Menos [31] is part of a multisite project coordinated by the World Health Organization. This self-help program is directed at persons with hazardous or harmful alcohol use and served as a model for the functionalities.

On the basis of the literature review of Web-based interventions, the specific characteristics of the PAADD were defined as follows:

1. The PAADD is a stand-alone intervention directed at persons with risky levels of drug use and not at those with drug dependence.

2. It offers strategies to address substance abuse and depressive symptoms together.

3. It includes 4 steps to establish a baseline and a treatment goal, in addition to strategies to change the pattern of use, stay focused on the goal, and prevent relapses.

4. It offers contact from a counselor, which previous studies have shown produces favorable results [44,45]

#### Stage 2. Validation of Content

Once the outline and the core contents for each step were planned, these were shared with a focus group of 8 experts in addiction treatment from the public mental health care system in the Mexico City metropolitan area. Participants were asked to comment on the proposed techniques and the possible impact of the program and to provide suggestions for improvement and implementation.

These experts expressed the opinion that the CBI strategies and techniques for changing substance use behaviors were appropriate. Their comments were useful for fine-tuning the inclusion criteria: (1) medium risk level of drug use according to the Alcohol, Smoking and Substance Involvement Screening Test [46] and (2) moderate depressive symptomatology assessed with the Patient Health Questionnaire (PHQ-9) [47] administered in a first face-to-face session. They suggested including different easy-to-use applications including workable user reports to record the amount of drugs consumed and sending users reminders to finish their activities. They also offered observations on the functions of the counselor, suggesting in particular that there be an initial face-to-face meeting in order to forge a therapeutic alliance, explaining that the PAADD is not a treatment in real time but that the counselor would provide written feedback. It was also suggested that training be offered to the counselor in skills such as empathy and motivational interviewing–based reflective listening. Finally, they offered recommendations regarding the vocabulary used so that the program would also be accessible to users with a low educational level.

#### Stage 3. Structure of the PAADD

**Figure 1:**
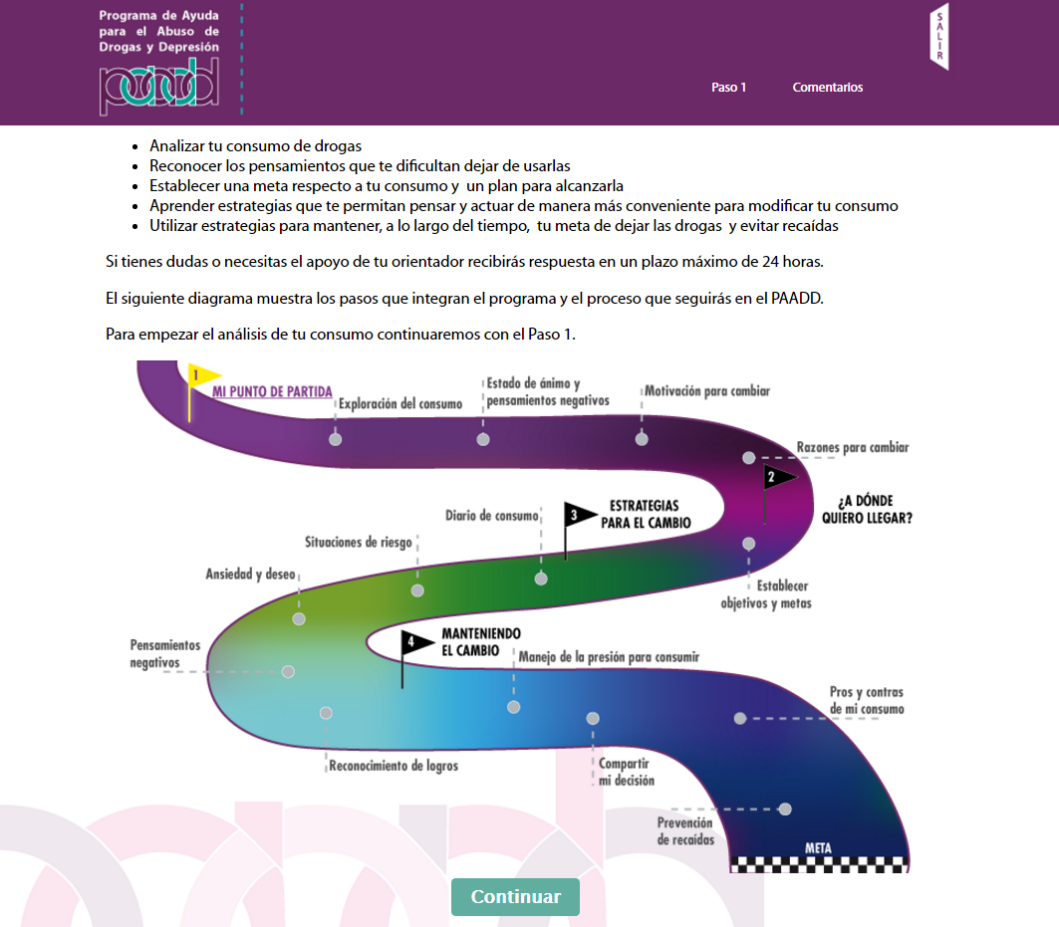
Help Program for Drug Abuse and Depression (Programa de Ayuda para Abuso de Drogas y Depresión, PAADD)'’s welcome page. Illustration of the stages and tools comprised in the program.

**Figure 2:**
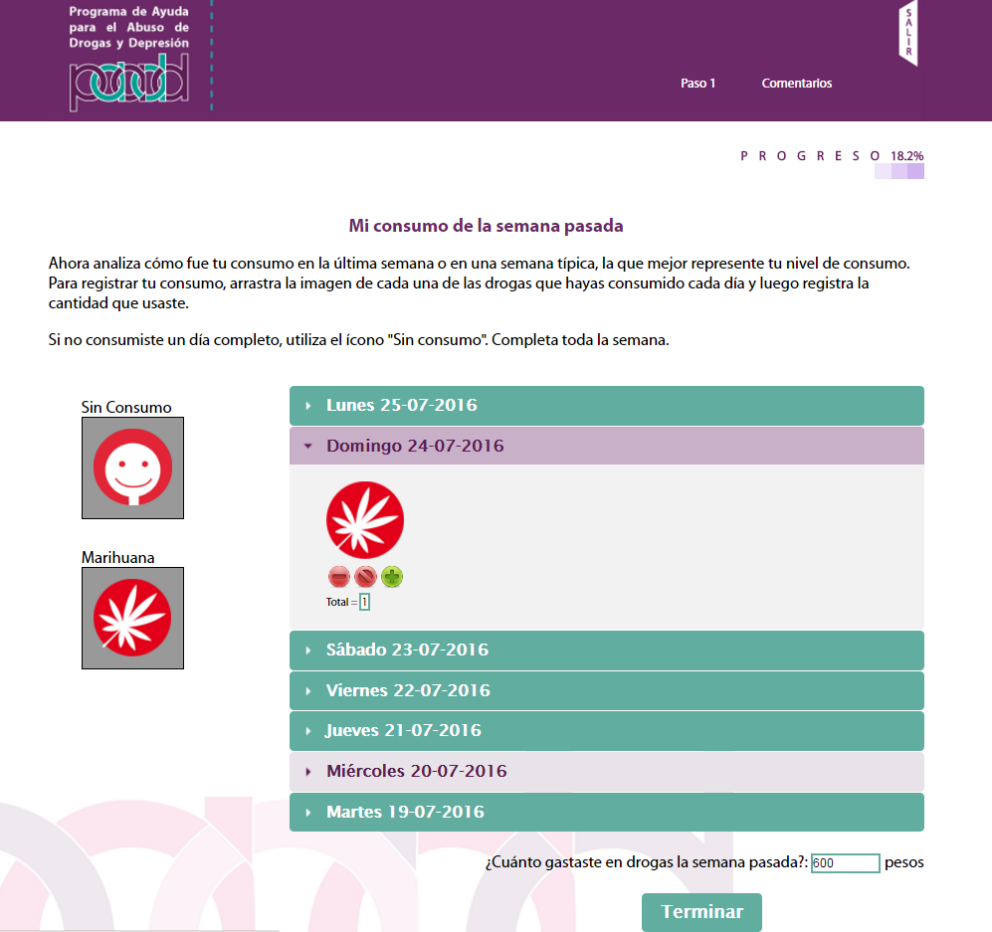
Timeline followback in step 1. Tool used to record drug use during the previous week and the amount of money expended.

**Figure 3:**
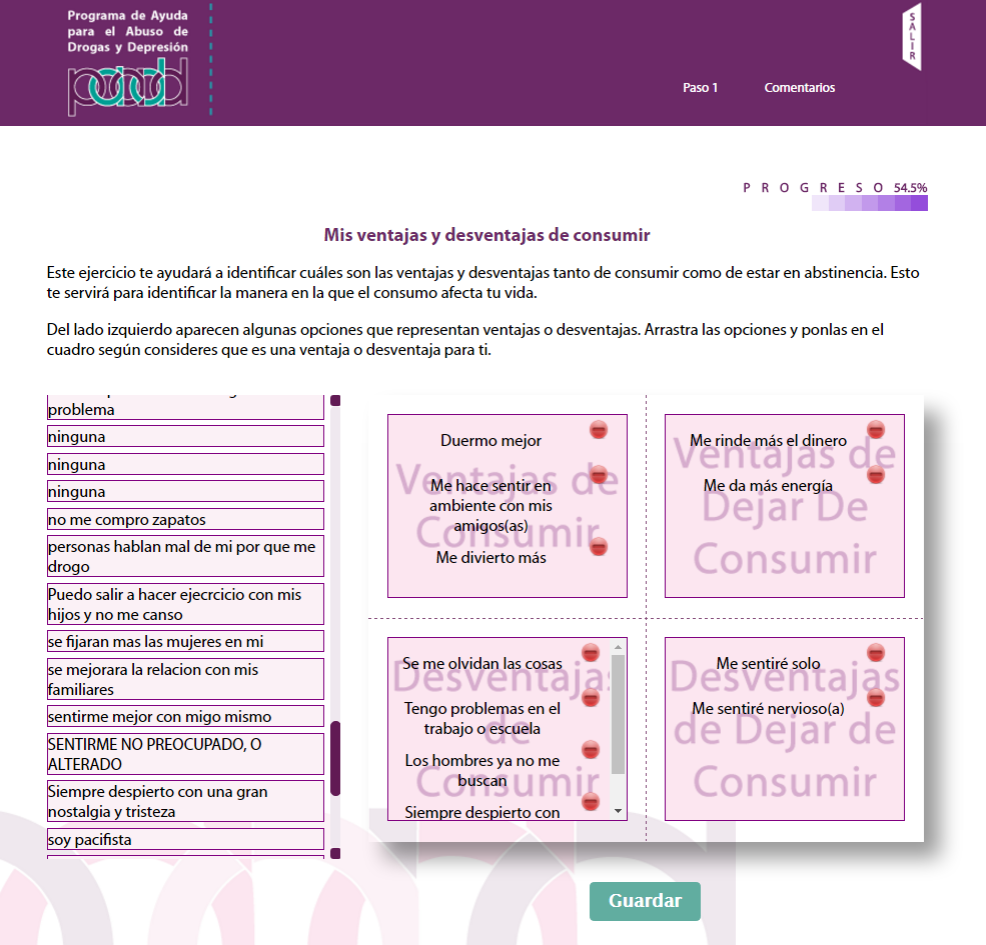
Decisional balance in step 1. Tool to identify advantages and disadvantages of drug use.

**Figure 4:**
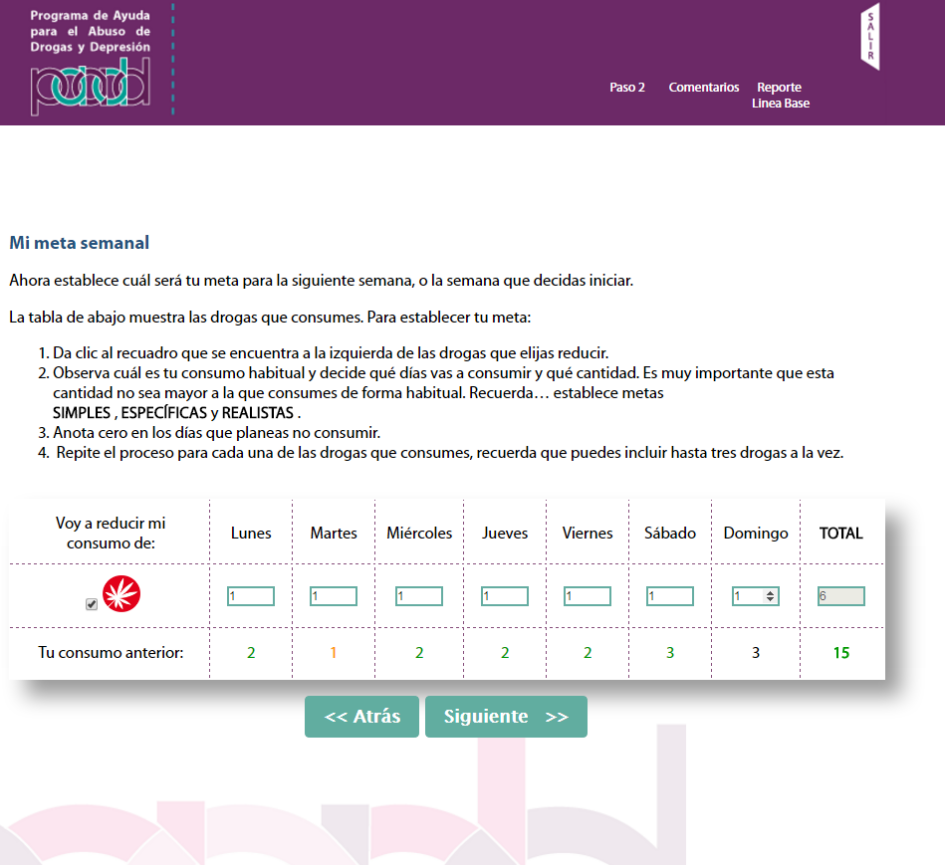
Goal setting in step 2. Tool to set a consumption goal for the following week.

**Figure 5:**
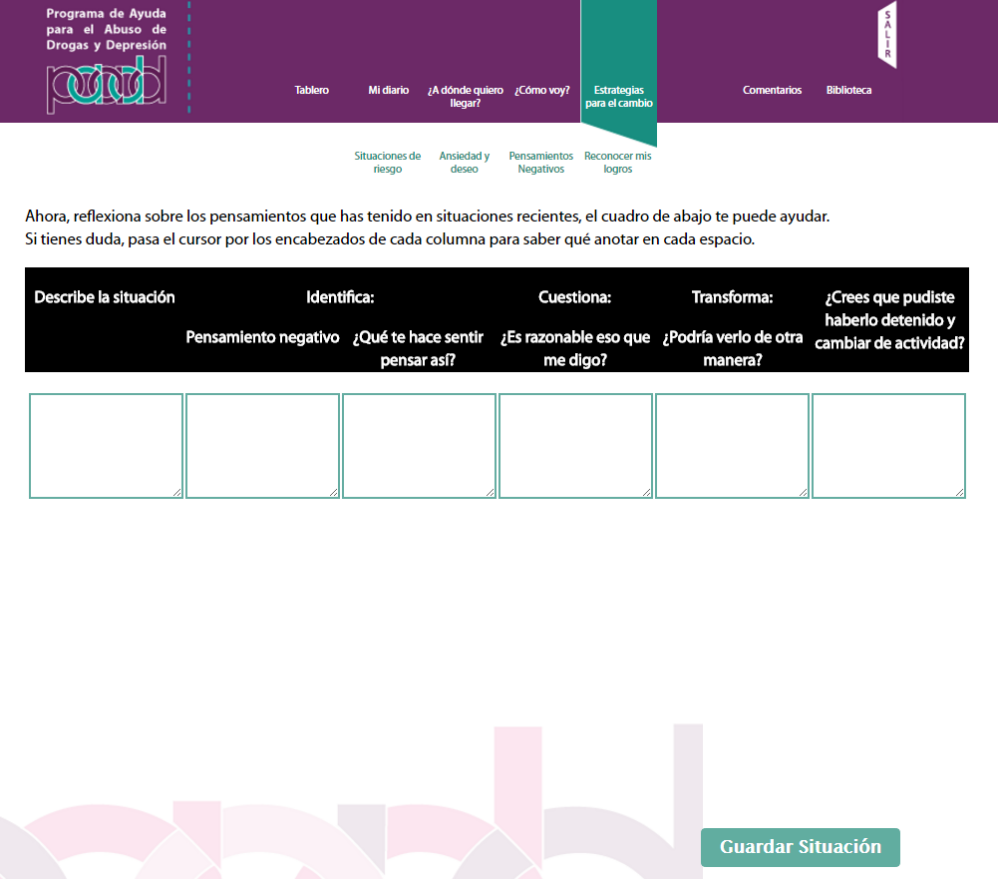
Cognitive restructuring in step 3. Tool to analyze negative thoughts associated with drug use.

The PAADD was structured into 4 successive steps. The home page is shown in Figure 1. Table 1 indicates the CBI strategies used in each step, such as self-control, functional analysis of consumption, and exercises to identify risk situations and transform negative thoughts associated with depressive symptoms; Figures 2-5 show some examples of the content and functions of each step. Users receive feedback from the counselor via a message system to motivate them to complete all the exercises.

**Table 1:** Overall structure of the Web-Based Help Program for Drug Abuse and Depression (Programa de Ayuda para Abuso de Drogas y Depresión, PAADD, in Spanish).

Step	Cognitive behavioral strategies
Step 1: My Starting Point	Establishment of baseline Identification of pattern of use Depressive symptoms Identification of negative thoughts Decisional balance Motivation and reasons to change
Step 2: Where Do I Want to Be?	Goal setting
Step 3: Strategies for Change	Self-monitoring Functional analysis of substance use Developing an action plan Psychoeducation Relaxation exercises Stopping unwanted thoughts Cognitive restructuring—link to ADep^a^ [31] Positive reinforcement
Step 4: Maintaining Change	Social skills for resisting pressure Seeking social support Assertiveness Monitoring results Adopting behaviors incompatible with substance use Relapse prevention

^a^ADep: Ayuda para Depresión (Help for Depression).

##### Step 1: My Starting Point

The aim of this module is to promote the awareness of personal drug use and its risks in order to increase the motivation to change. This module includes assessment instruments and behavioral strategies to establish a baseline. At the end, users receive a printable report and are asked if they are committed to participating in a process of change. A positive answer directs them to step 2; a negative answer redirects them to a feedback page to prompt reflection about the consequences of drug use.

##### Step 2: Where Do I Want to Be?

The purpose of this step is to set treatment goals. Users are provided with various tools, such as the recommendation to define a goal (simple, specific, realistic), to establish a reduction plan (to focus on 1 drug at a time, to plan ahead, to remember that any reduction in use is a step forward), and to set a time limit to achieve the goal. At the end of this step the user receives a printable feedback page.

##### Step 3: Strategies for Change

Step 3 is aimed at achieving behavioral change in accordance with the goals established in step 2. Users are advised to log in every day, record daily drug use, review the progress graph, and do an activity to achieve the change. They are offered tools to perform a functional analysis of substance use behavior, to develop action plans to face risk situations, to apply emotional control techniques to cope with anxiety, to generate positive self-reinforcement, and for cognitive restructuring of negative thoughts.

##### Step 4: Maintaining Change

The objective of step 4 is to reinforce changes in drug use behavior and avoid relapses. It offers strategies for recognition of success (self-reinforcement), for facing pressure to use drugs (assertiveness), and for seeking social support, among others. The counselor makes the decision to enable this step in accordance with the results from step 3.

The program is designed to be completed in 8 weeks, but this period can be extended if necessary depending on the evaluation of the counselor, who might suggest specific activities that the participant may have skipped, according to the progress and needs of each user. The role of the counselor also includes monitoring the completion of the activities as well as providing written feedback and encouragement within; all these functions are performed through a messaging system within the program within 24 hours after a user logs in. This is a minimal contact scheme that differs from conducting therapy sessions via the Internet and represents an opportunity to optimize time because one counselor can monitor 2 or more patients simultaneously.

The structure of the Web-based intervention was outlined as a diagram in order to establish the sequence of the steps involved. The PAADD was constructed by a commercial programming company as a Web application accessible through computers, laptops, or tablets with any browser; although it is not a mobile application, it can also be accessed with a mobile phone. It took into account 3 types of end users: administrators, counselors, and substance users. Functionality and usability tests were performed on the completed application.

#### Stage 4. Functionality Test

The purpose of this process was to ensure that the PAADD met the technical requirements set out in the functional design and to identify programming glitches. The test specifically verified the following:

- The program worked according to specifications.
- The database responded to the input and output specifications of expected information.
- The system followed the sequence identified in the functional design.

These functionalities were tested by members of the research team (n=7), who interacted with the program and verified each one, following a checklist. Errors found were corrected and a second test was performed. This functionality testing was then followed by a usability test.

#### Stage 5. Usability Test

Usability is defined as the ability of a software or program to be understood, learned, used, and be attractive to the end user under specific conditions of use [48]. The objectives of this phase were (1) to test the usability of the PAADD and (2) to identify the sections that needed adjustment to improve users’ experience.

### Participants

A total of 9 health professionals from Mexico City, State of Mexico, and the states Morelos, and Baja California agreed to participate. Inclusion criteria were that (1) they worked in an addiction treatment center; (2) they had at least a year of experience working in substance abuse treatment using cognitive behavioral strategies; (3) they had experience using the Internet; and (4) they were regular email users. Each was asked to invite patients under treatment (n=20) to give their opinion of the PAADD. The inclusion criteria for the patient group were that (1) they were adults; (2) they had received a minimum of 4 treatment sessions for abuse of psychoactive substances during the previous 6 months; (3) they could read and write; (4) they had experience using the Internet; and (5) they were regular email users.

### Evaluation Instruments

All participants (both health professionals and patients) responded to a questionnaire about their Internet usage and received an instruction guide for usability test. The checklist for the health professionals focused on evaluating the role of the counselor (usefulness of strategies, contents, and message system), whereas that for patients focused on their perspective about language and ease of use.

### Procedure

This procedure was approved by the Research Ethics Committee of the Ramón de la Fuente Muñiz National Institute of Psychiatry. All participants were volunteers and signed an informed consent form with the understanding that the data might be used in research. All information obtained in this program is confidential and available only to members of the research team. The questionnaires, forms, and other documents were identified by a numeric code that avoids the identification of participants.

The usability test was based on a selection of tasks and pages according to the guidelines proposed by Dumas and Loring [49]: (1) frequently used tasks; (2) tasks that are basic to the general program; (3) tasks that are critical because they affect other parts of the design, even though they may not be frequently used; (4) tasks in which problems are anticipated; (5) tasks that test the structure and components of the design; and (6) the time available for the evaluation.

The tests carried out by the health professionals consisted of navigating the PAADD, following a list of specific actions, and reporting on their experience. The tests by patients were carried out at the centers where they were receiving treatment. A member of the research team accompanied them and recorded their commentaries and suggestions.

## Results

Results of the usability test are presented here separately by type of participant.

### Characteristics of the Health Professionals

The group included 5 women and 3 men with an average age of 30.25 years (SD 5.33). All participants had a master's degree in psychology of addiction and had an average of 5 years of experience using the CBI approach to treat persons with problems of substance abuse. All participants were regular users of the Internet, with an average daily use of 6.1 hours, the majority from their home and workplace.

### Professionals’ Suggestions

The main suggestions from the health professionals focused on the content and format but not on the structure of the program, for instance, recommendations to change the color scheme, page layout, font size, arrangement of functions, inclusion of confirmation messages at the end of activities, avoiding the use of technical language and “read more” links, providing short introductory texts for every task, and creating more visible help buttons with specific explanations and tips. They found the functionalities easy to learn, although they indicated that some were not intuitive at first glance. They suggested providing feedback about every activity and including more activities and exercises within the program, as well as more tips on how to end substance abuse. They also suggested the possibility of including testimonials from persons who had benefited from the program (see Table 2).

### Characteristics of the Users

The user group included 16 men and 4 women, 60% of them single, with an average age of 29.6 (SD 9.1) years. In this group 70% (14/20) had completed high school, 15% (3/20) an undergraduate degree, and 15% (3/20) elementary school; 65% (13/20) were employed, 20% (4/20) did not work, and 15% (3/20) were students. All participants were regular users of the Internet, with an average daily use of 3 hours, the majority from home (85%) and the rest using their cell phone and/or at Internet cafes.

### Users' Suggestions

Users suggested a more dynamic design with images and colors, clearer, more concrete instructions, inclusion of empathetic, reflective feedback, and also a chart showing the names and appearance of different drugs. The majority said it was important to be able to modify the weekly goal and clarify the sequence of activities in the program (see Table 3).

The observations and suggestions of users and health professionals were compiled into a document that served as a guide for development of a new version of the PAADD, which was in turn put through an evaluation process.

**Table 2:** Observations and suggestions from health professionals.

Step	Positive aspects	Negative aspects	Suggestions
Step 1	Includes counselor from first contact. The format of the instruments facilitates response. Providing a summary of results of the initial evaluation favors a decision to change.	It is necessary to clarify some instructions and the sequence of the instruments. Feedback is very brief. Cannot log fractional amounts of substances used.	Indicate what each instrument evaluates. Provide motivational, reflective, and personal feedback. Include a list of common names for drugs. Allow logging of open responses. Include interactive applications, examples, and vignettes.
Step 2	Gives users the responsibility to set their own goals. Weekly goal report helps the user not to forget it. Information provided helps users to plan their weekly goals.	The sequence of activities is not clear. The setting of goals does not consider multiple use.	Provide simpler instructions. Compare habitual use with the weekly goal. Allow setting of goals for more than 1 drug.
Step 3	The graph of use allows users to observe their changes. The diary of drug use allows the identification of risk situations. The summary of drug use situations allows for the analysis of factors that interfere with the goal.	The sequence of activities is not clear. Complex texts. There is no indication to show the end of the activities.	Make the sequence of activities explicit. Include clear, concise texts with interactive elements such as vignettes and images. Include a brief explanation of the significance of the graphs. Add a phrase to show when an activity is finished.
Step 4	The exercises included are strategically important to maintain the change in drug use. They are easy to do.	Needs automatic feedback on finishing exercises. Needs an indication of the end of an activity.	Add a phrase to show when an activity is finished.

**Table 3:** Observations and suggestions from drug users in treatment.

Step	Positive aspects	Negative aspects	Suggestions
Step 1	The initial information and the informed consent help to understand what the program is about. The feedback for every questionnaire is very useful. The report lets you see the combined results of the evaluation.	The design of the program is very serious. The exercises on advantages and disadvantages of drug use are complex. The instructions for some questionnaires are not clear. There is technical language in the instructions, exercises, and feedback.	Use a dynamic design that includes images, vignettes, and more color. Provide clear and simple instructions. Give reflective and empathetic feedback to help interpret the result. Include examples of different forms and names of drugs. Highlight the link to the page on risks associated with drug use.
Step 2	Feedback on setting goals helps to remember them.	The instructions are complex. The sequence of activities is not clear.	Include more than 1 drug in the log of goals. Allow a change in goal. Compare the weekly goal with habitual use logged in step 1.
Step 3	The initial screen shows a general overview of the activities of this step. The drug use diary lets you identify the factors that encourage use. The graphs show progress in reducing drug use and situations of use and nonuse.	The instructions are complex. The diary does not allow logging of fractional amounts of substances used. Interpretation of the graphs is not clear. Texts are long. Needs an indication of when all the activities are finished.	Provide clear and concise instructions and interactive examples of the activity log. Provide more concrete texts. Include a brief explanation of the meaning of the graphs. Make the sequence of activities explicit. Include a phrase that shows the end of the activity.
Step 4	The activities are easy to do.	Activities do not provide feedback, and there is no indicator of when they are finished.	Give automatic reminders in case the weekly goal is not met. Indicate a time period for each activity.

## Discussion

### Main Findings

This paper describes the development and results of the usability test of PAADD, a Web-based intervention with the participation of a counselor, the first of its kind in Latin America that is designed to reduce substance use and depressive symptoms. The development of the program was systematic and rigorous. It began with a review of the literature on brief interventions and incorporated the opinions of experts in addiction treatment as well as 2 types of end users, following a procedure similar to that reported by Morrison et al [50]. The result of the process was a Web-based program that contains the key elements of a CBI, with a user-friendly design, simple instructions, and intuitive and time-efficient functions.

The translation of cognitive behavioral strategies to the concrete activities of the PAADD was complex and time consuming. Van Voorhees et al [51] note that a focus on detail is necessary to achieve good results in this type of process. Likewise, a multidisciplinary approach considering different points of view leads to more satisfactory results [52]. The detailed description of the process of designing a Web-based program is an important contribution, given that there is no specific method for the creation of this intervention modality [50]. This study can therefore serve as a guide to others interested in this field.

### Acceptance and Usability Test

An important characteristic of the PAADD is that it includes the participation of counselors who provide feedback according to the motivational interviewing techniques. This element is intended to strengthen adherence, as it has been shown that the inclusion of health professionals has a positive influence on retention and motivation of participants in such programs [53,54].

In the usability test, drug users commented that the PAADD was professional and clear. Many felt that the information presented within the modules was relatively easy to follow, comprehensive, and of good quality. A few participants reported at times feeling impatient to receive the next module. The majority of users commented positively on the content of the program and felt that they could benefit from it. In their view, it helped principally with improved self-awareness of their pattern of drug use. They reported that the PAADD encouraged them to think about self-management techniques, how to monitor their thoughts and feelings, and how to regulate their behavior and consumption patterns. These observations suggest that the design process was successful; it resulted in a program that is easily usable by persons who wish to end their substance use and who present depressive symptomatology.

The PAADD may have advantages over traditional face-to-face therapies because of its low cost, wide accessibility, anonymity, and independence of time and distance factors. It may be a good alternative for persons not receiving treatment owing to factors such as physical distance from available services, lack of qualified providers, socioeconomic condition, and stigma, although these potential benefits will have to be verified in specific studies.

### Future Directions

In future efforts it will also be important to consider that the initial investment for the design of a Web-based intervention can be high and that it is necessary to perform a cost-benefit analysis to document the viability of the project. It should also be kept in mind that technologies are developing rapidly; in order to provide increased availability and accessibility, it is necessary to plan from the beginning the possibility of using the program on diverse types of devices, such as mobile phones and tablets, and not just on computers.

Because PAADD is not an open-access program, it will also be necessary to design a dissemination strategy that would include training of counselors and to promote the use of PAADD in specialized treatment centers. This will allow to document adoption and implementation in real-life scenarios.

### Limitations

The lack of forums or chat functionalities for users to interact with each other may be a limitation in this program because having contact with others in the same situation can be a source of support. It is also possible that the small number of users participating in the usability test limited the number of opinions regarding improvements to the program. Such limitations have been reported in similar studies [53,54].

### Conclusions

Data from the usability test indicate that the PAADD has the necessary features to support users in their process of reducing drug use. If this is corroborated in effectiveness studies, the program could be used as an alternative to treatment as usual.

## Abbreviations

ADep: Ayuda para Depresión (Help for Depression)
CBI: cognitive behavioral intervention
PAADD: Programa de Ayuda para Abuso de Drogas y Depresión (Web-based Help Program for Drug Abuse and Depression)
PHQ-9: Patient Health Questionnaire
